# Advances in the Detection of Toxic Algae Using Electrochemical Biosensors

**DOI:** 10.3390/bios10120207

**Published:** 2020-12-16

**Authors:** Linda K. Medlin, Maria Gamella, Gerardo Mengs, Verónica Serafín, Susana Campuzano, José M. Pingarrón

**Affiliations:** 1 Marine Biological Association of the UK, The Citadel, Plymouth PL1 2PB, UK; 2 Department of Analytical Chemistry, Universidad Complutense de Madrid, 28040 Madrid, Spain; mariagam@quim.ucm.es (M.G.); veronicaserafin@ucm.es (V.S.); susanacr@quim.ucm.es (S.C.); pingarro@quim.ucm.es (J.M.P.); 3 Ecotoxilab, 28550 Madrid, Spain; gmengs@ecotoxilab.com

**Keywords:** toxic algae, biosensors, barcodes, early warning system

## Abstract

Harmful algal blooms (HABs) are more frequent as climate changes and tropical toxic species move northward, especially along the Iberian Peninsula, a rich aquaculture area. Monitoring programs, detecting the presence of toxic algae before they bloom, are of paramount importance to protect ecosystems, aquaculture, human health and local economies. Rapid, reliable species identification methods using molecular barcodes coupled to biosensor detection tools have received increasing attention as an alternative to the legally required but impractical microscopic counting-based techniques. Our electrochemical detection system has improved, moving from conventional sandwich hybridization protocols using different redox mediators and signal probes with different labels to a novel strategy involving the recognition of RNA heteroduplexes by antibodies further labelled with bacterial antibody binding proteins conjugated with multiple enzyme molecules. Each change has increased sensitivity. A 150-fold signal increase has been produced with our newest protocol using magnetic microbeads (MBs) and amperometric detection at screen-printed carbon electrodes (SPCEs) to detect the target RNA of toxic species. We can detect as few as 10 cells L^−1^ for some species by using a fast (~2 h), simple (PCR-free) and cheap methodology (~2 EUR/determination) that will allow this methodology to be integrated into easy-to-use portable systems.

## 1. Introduction

In the last century, human poisonings by harmful algae have occurred most often because regular monitoring programs did not exist worldwide. It is now compulsory for all European countries with a coastline to have national monitoring programs for toxic marine algae. Over time, more toxins have been added to the regulatory panel of toxins that must be monitored before shellfish are released for sale. Today, cases of human poisonings are rare and occur when local aquaculture farms violate national health authorities’ regulations that close harvesting areas and forbid seafood commercialization or when shellfish harvesting is done locally by the public because there are no signs posted forbidding them to do so. 

Harmful algal blooms (HABs) are becoming more frequent as climate changes, with tropical toxic species moving northward. This is especially true for the Iberian Peninsula. The taxa that are moving northward are the benthic/epiphytic dinoflagellates belonging to *Gambierdicus, Coolia, Ostreopsis* and *Prorocentrum lima.* The seafood-borne illness ciguatera, produced by the toxins from *Gambierdiscus,* is characterized by gastrointestinal, neurological and cardiovascular symptoms. Recently, these species have been reported in new subtropical and even temperate geographical areas because climate change has led to the expansion of ciguatoxins (CTXs) producing dinoflagellates, as well as to the globalization of the fishing industry. 

The first report of the occurrence of *Gambierdiscus* sp. as a potential producer of ciguatoxin in Europe was from Crete in 2003 [1]. Latter in Madeira, suspected ciguatera poisonings were reported from fishermen who consumed fish contaminated with CTX toxins [2]. The surveillance system, SVEICC (Sistema de Vigilancia Epidemiológica de la Intoxicación por Ciguatera en Canarias, Epidemiological Surveillance System of Ciguatera Poisoning in Canary Islands), was implemented in 2009 making it compulsory to notify the island’s health authorities of any case where ciguatera symptoms were suspected [3,4]. What is more worrying is that poisonings caused by ciguatoxin come from ingesting either shellfish or molluscs, whereas most other toxic algal problems come only from ingesting molluscs because the entire animal is eaten. [5]. 

Palytoxins (PTXs) are relatively new toxins [6], even causing fatalities in humans. They were first identified in soft corals of the soft coral genus Palythoa, who gave their name to the toxins. They are also found in the benthic dinoflagellate genus, *Ostreopsis (Ostreopsis siamensis, O. mascarenensis, O. ovata*), and in the cyanobacterium, *Trichodesmium*. PTXs were first reported in warm waters where soft corals grow naturally, but they seem to have spread into temperate waters [7,8] where *Ostreopsis* spp. occur. *Ostreopsis* blooms (mostly cf. *ovata*) have also been reported in France, Greece, Italy, Spain and Croatia [9,10,11]. A recent bloom of *Ostreopsis* spp. on the Algarve coast of Portugal) produced palytoxin analogues, e.g., ovatoxins. This suggests that *Ostreopsis* may be spreading from the Mediterranean Sea into the North Atlantic [12]. Besides its health impact, *Ostreopsis* blooms have a negative economic impact on the tourism industry. France, Italy and Spain have alerts only for tourism because of the negative effects of PTX aerosols on humans. Beaches can be closed for swimming; however, there are no closures for aquaculture.

PTXs and CTXs are considered to be emerging marine toxins in the European Union because they have appeared with increasing frequency and are the key targets in the EU project EMERTOX. They are not yet listed among the toxins required for monitoring. Monitoring programs detecting the presence of toxic algae before they bloom, are of paramount importance to protect aquatic ecosystems, aquaculture, human health and local economies. The goals of EMERTOX are to produce novel sampling methods for these benthic dinoflagellates and novel methods for sampling for their toxins. Making rRNA probes falls into the remit of novel sampling methods with the goal of making a rapid low-cost detection system that can be implemented as an early warning system. Our choice for a rapid low-cost detection system is a laboratory on a chip (LOC).

Rapid and reliable species identification methods using molecular barcodes coupled to biosensor detection tools have received increasing attention over the past decade as an alternative to the legally required but impractical standard microscopic counting-based techniques. We present here probes for the benthic dinoflagellate species belonging to *Gambierdiscus, Ostreopsis, Coolia* and *Prorocentrum lima,* which are species moving into European waters as climate changes. These probes are not included in the MIDTAL microarray for toxic algae. These new probes have been exploited in connection with recently reported designs of sandwich or direct hybridization assays and enzymatic labeling using the enzyme horseradish peroxidase (HRP) implemented on the surface of streptavidin (Strep)-MBs [13,14,15] for target DNA or RNA determination followed by amperometric detection at SPCEs upon magnetic fixation of the MBs bearing the HRP-labeled duplexes using the system H_2_O_2_/HQ. The use of MBs as solid supports in electrochemical biosensors avoids the need to apply and optimize laborious protocols for modifying electrochemical substrates leading to advantageous biodevices in terms of assay time, sensitivity and minimization of the sample matrix. The variation in the cathodic current, attributed to the enzymatic reduction of H_2_O_2_ mediated by HQ is proportional to the concentration of the target DNA/RNA and thus proportional to the number of target cells in the water, hence our early warning system.

## 2. Material and Methods

**Figure 1:**
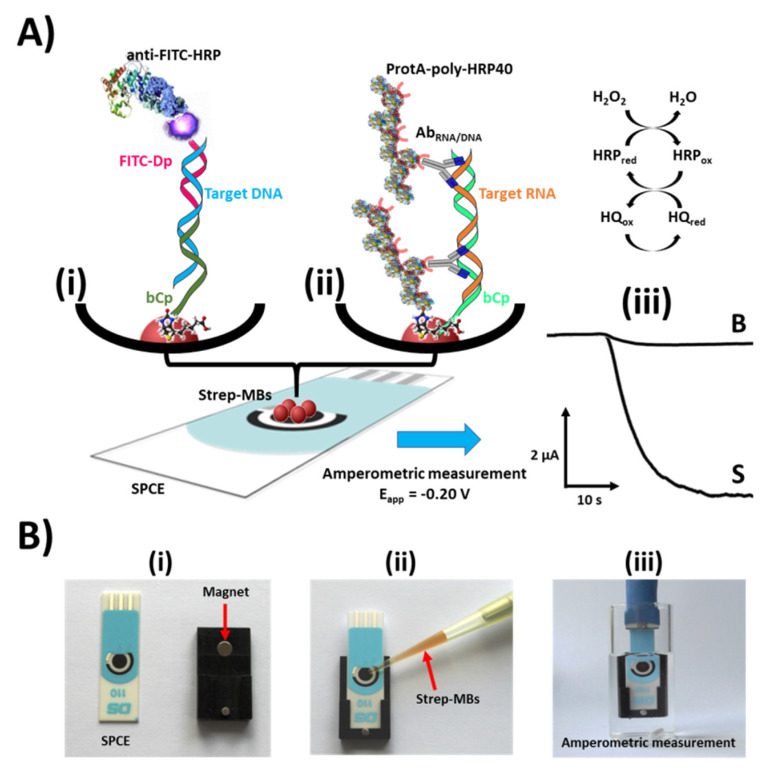
(**A**) Schematic diagram of the sandwich hybridization (**Ai**) with two probes (grey and red) and the heteroduplex hybridization (**Aii**) with one probe (green) immobilized on an electrode and concomitant redox reaction to generate the electrochemical signal (far right). Real amperometric traces obtained with the developed biosensors in the absence (Blank signal, B) and in the presence of a representative algae RNA (Signal, S) (**Aiii**). (**B**) Photographs showing the magnetic capture of the magnetic microbeads (MBs) on the working electrode of a screen-printed carbon electrode (SPCE) (**Bi**,**ii**) and the immersion of the SPCE/PMMA casing ensemble into the electrochemical cell where amperometric detections are carried out (**Biii**).

**Figure 2:**
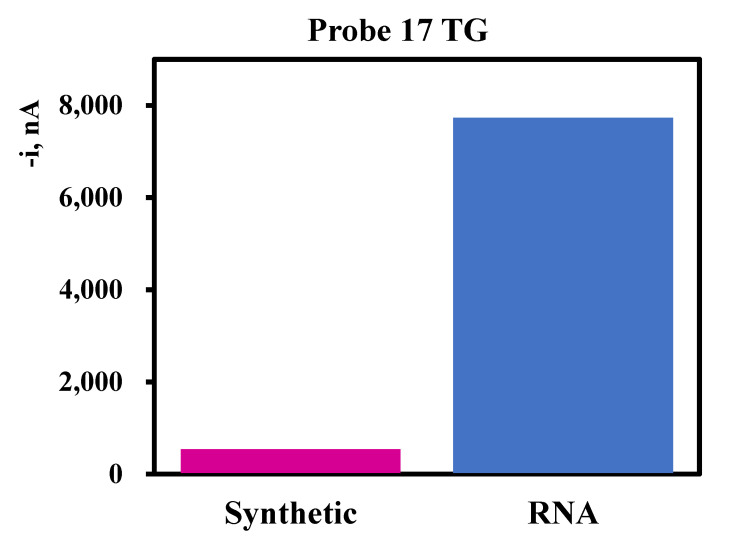
Comparison of the 20-fold difference in signal intensity between real RNA and synthetic target DNA for 100 pM concentration.

*Probe Design:* We designed probes or barcodes from the SSU or LSU genes to recognise each of the emergent toxic species targeted in EMERTOX using the probe match function in the ARB program [16]. Probes were designed to 50–60 nts in length so that the melting temperature was close to 60 °C. This meant that the G/C content was at least 50%. Of the many probes designed by the ARB probe match function, we chose only a single probe to test and that probe usually had more than two mismatches to other species in our database. The mismatches were positioned in the middle of the probe to facilitate destabilization of the helix. Following probe design, we blasted the probes to Genbank for a comparison with the most up to date sequence database to verify probe specificity in-silico. A total of 53 probe sets (protected by patent PCT/DE2003/002124) were designed for the sandwich hybridization method) for the species moving into European waters (*Gambierdiscus, Ostreopsis, Coolia* and *Prorocentrum lima*) (Table 1). Each set contains the following two probes plus their target: (1) capture and (2) signal DNA probes that bind to (3) a synthetic DNA target (Figure 1A(i)). Because we did not have RNA from all species immediately, we used the sandwich hybridization with synthetic targets to test probe specificity. Real RNA can be about 20× more sensitive than synthetic target (Figure 2). Once RNA was obtained and the concentration and purity checked (Nanodrop One, from Thermo Scientific), we made calibration curves to convert signal intensity to cell numbers and determine the limit of detection. To detect RNA targets we used only a single synthetic DNA probe, which was a combination of the capture and signal probe with a biotin 5’ label (Figure 1A(ii)).

The probes were designed to hybridise between 50–60 °C and were synthesized by CondaLab (Madrid, Spain), with a 5’ biotin label on the capture probe and a 3’ fluorescein isothiocyanate (FITC) label on signal probe. Synthetic targets were unlabelled and were complementary to the combined capture and signal probes as DNA. Potential cross-reactivity was checked using the program ChipCheck [17] (Table 2).

*Probe Immobilization:* 5 μL of the Streptavidin-MBs suspension (Strep-MBs, 2.8 μm Ø, 10 mg mL^−1^, Dynabeads M-280 Streptavidin, 11206D, from Dynal Biotech ASA) were aliquoted into a 1.5 mL microcentrifuge tube, washed 2× with 50 μL of B&W (pH 7.5) buffer (10 mM Tris–HCl solution containing 1 mM EDTA and 2 M NaCl), and incubated for 60 min (37 °C, 950 rpm) in 25 μL of 0.1 μM capture probe (40-mer) solution prepared in B&W buffer, pH 7.5.

*Target hybridization:* Subsequently, the supernatant was removed and washed 2× with 50 μL of a commercial casein blocker solution (a ready to use PBS solution containing 1% *w/v* of purified casein, from Thermo Scientific). The capture probe-MBs were incubated for 30 min (55 °C, 950 rpm) in 25 μL of a solution containing the synthetic target (DNA) supplemented with the FITC-modified signal probe (0.25 nM) or RNA for each algal species. Concentrations of target DNA/RNA ranged from 1, 10, 100 and 1000 pM were used to test the sensitivity of the probes and if they were saturated on the MBs. Cross-reactions to non-targets were tested only with the synthetic ones at 100 pM. To detect target RNAs, they were denatured at 92 °C (in a thermocycler SensoQuest LabCycler, Progen Scientific Ltd.) for 7 min in PBS buffer and kept on ice until their incubation at 55 °C with capture-signal probes as a single probe-MBs conjugate. Thereafter, the supernatant was removed and two additional washing steps with 50 μL of casein blocker solution were carried out.

*Target Recognition:* The selective recognition of the FITC-labelled sandwich DNA/DNA heterohybrids captured onto the MBs and their enzymatic labelling were performed in a single step by incubating the modified MBs for 30 min (37 °C, 950 rpm) in 25 μL of a mixture solution, prepared in casein blocker solution, containing anti-FITC Fab fragments conjugated with HRP (anti-FITC-HRP, 2 μg mL^−1^) from Roche Diagnostics GmbH (Figure 1A(i)). Thereafter, the supernatant was removed, washed 2× in casein blocker solution and resuspended in 50 μL of 0.05 M sodium phosphate buffer solution (pH 6.0) before electrochemical detection. For the detection of total RNA with the single DNA probes, their enzymatic labelling was performed in a single step by incubating the single capture-signal probe-MBs for 30 min (37 °C, 950 rpm) in 25 μL of a mixture solution, prepared in casein blocker solution, containing 2 μg mL^−1^ of a commercial RNA-DNA hybrid antibody (Ab_RNA/DNA_, clone: D5H6, from Covalab) and a commercial bacterial antibody binding protein (ProtA) conjugated with a homopolymer containing 40 HRP molecules (ProtA-poly-HRP40, dil. 1/50, from antibodies-online) (Figure 1A(ii)).

It is important to point out here that the bioassay and enzymatic labelling strategies are based on previous results obtained by the group of Pingarrón and Campuzano in the exhaustive comparison of different strategies for the development of highly sensitive MBs-based electrochemical nucleic acid biosensors [13] and, that these biodevices have already demonstrated potential applicability for PCR-free electrochemical biosensing of animal or plant-food derived nucleic acids in raw mitochondrial [14] and genomic [15] DNA extracts.

*Amperometric measurements:* The amperometric measurement was carried out in phosphate buffer solution (pH 6.0) containing 1 mM hydroquinone (HQ, from Sigma-Aldrich from Madrid, Spain) at SPCEs (DRP-110, from Methrom-DropSens) upon magnetic capturing of the resultant MBs onto the working electrodes of the SPCE previously placed in a custom polymethylmetacrylate (PMMA) casing with an embedded neodymium magnet (Figure 1B(i,ii)) and connected to the potentiostat (CH Instrument model 812B controlled by software CHI812B from Austin, TX, USA) through the specific connector cable (DRP-CAC, also from Methrom DropSens). The ensemble was immersed into an electrochemical cell containing 10 mL of 0.05 M phosphate buffer (pH 6.0) supplemented with 1.0 mM HQ (Figure 1B(i–iii)), which is kept under constant agitation. A −200 mV vs. Ag/AgCl potential was applied. Once the current was stable, a 50 µL aliquot of 0.1 M hydrogen peroxide (30%, *w/v*, from Sigma-Aldrich) solution was added and we waited until a steady state was reached again to stop the reaction.

*Calibration Curves:* In those cultures where RNA was available, we performed calibration curves using the following RNA concentrations: 1, 10, 50 and 100 pM, occasionally 500 or 1000 pM. By converting the ng μL^−1^ concentration of the RNA to pM and using the total number of cells in the culture harvested, we calculated the number of cells equivalent to 1 pM of RNA (Table 3). Regression fits for the calibration curves are found in Table 4.

## 3. Results and Discussion

Of the 53 probes designed for emergent species, 20 have been synthesized and 17 tested for cross-reactivity in-silico and in-vivo to date because these probes are most relevant to the goals of the EMERTOX project and target European species/strains/clades that are known to be present or moving into in European coastal waters and in the Mediterranean. For *Gambierdiscus*, we tested probe specificity for *G. silvae*, *G. excentricus*, and *G. australis,* which are now commonly found in the Canary Islands [22]. Two other species/morphotypes (*G. caribaeus* and *G. carolinianus*) have been rarely seen with no surviving cultures and so were not tested. For *Coolia, C. monotis* and *C. tropicalis* and *C.* cf. *canarienesis* [23] were tested. The first species has been reported from Cape Verde (Emertox, unpubl.) and the last from the Iberian Peninsula [23]. *C. canarienesis* was not tested because we were unable to locate any cultures from this species. In our ARB tree, there were multiple clades of *Ostreopsis*, mostly unidentified. In European waters, toxic species of *Ostreopsis* include *lenticularis*, cf. *ovata*, and cf. *siamensis* [24]. The correct identification of *Ostreopsis* species with light microscopy is often problematic because *Ostreopsis* species are morphologically plastic and difficult to identify without molecular analyses [20]. Many identifications are referred to as “cf.” of a known species, usually *ovata*. Because of this uncertainty in species identification, we choose to design and test a genus-level probe. *Prorocentrum lima* is another paraphyletic taxon, with at least four major clades recovered in its phylogenetic analyses with nine or more subclades [25,26]. Not all of the species from the Zhang et al. study [25] were included in the Nishimura et al. study [26] but in our ARB database, all are included and we identified eight major clades for which we designed probes. We tested those clades that contained European sequences.

The remaining non-synthesized probes represent species/clades resident in other areas of the world and would be needed for a universal laboratory on a chip (LOC) along with the other species/clades are represented on the MIDTAL microarray. It is our long-term goal to make a LOC with the probes arranged in a microarray fashion but with an electrochemical detection rather than a fluorescent one. LOCs offer the most cost-effective and sensitive detection system for detecting target RNA in the environment.

In-silico tests involved using Chipcheck to calculate the energy needed to denature any helix, i.e., the binding of the probe to any target or non-target region. Probe binding to target resulted in a high value, whereas probe to non-target resulted in a low value. Potential cross-reactions between probe and non-target resulted in medium values closer to the values of the target.

**Figure 3:**
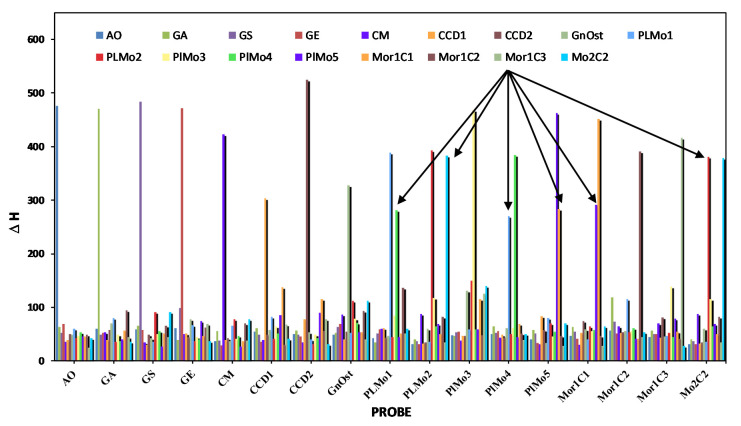
Plot of the enthalpy values needed to denature pairs of probes and targets.

We have demonstrated the potential cross-reactivity of any of our probes graphically in Figure 3 and quantitatively in Table 2. The numbers marked in red in Table 2 indicate the absolute energy needed to disassociate the DNA/DNA helix of probe and target. All probes, except probes for clades of *P. lima,* showed in-silico a high specificity for the target probe. Several arrows in Figure 3 mark probes that are showing potential cross-reactivity among the *P. lima* probes. We either renamed or redesigned those probes. In *P. lima*, the empirical potential for cross-reaction was evident in most of the probes and at least one of the probes (probe set 39) was redesigned as probe set 76 before testing began.

**Figure 4:**
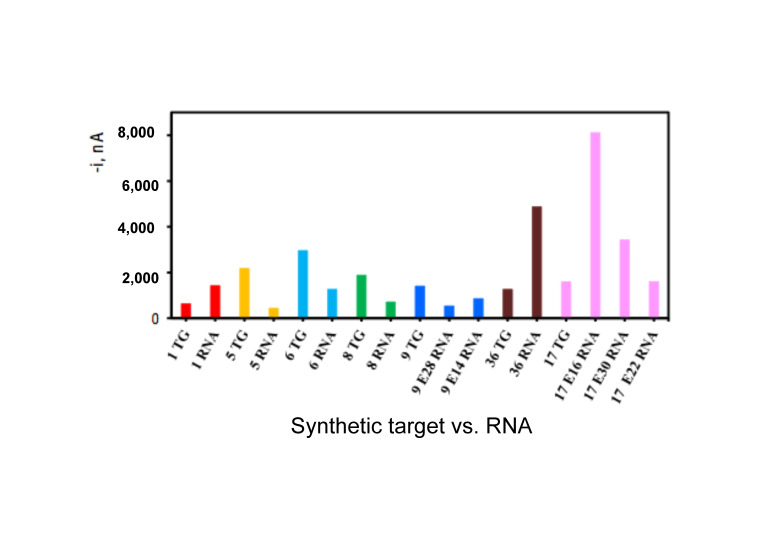
Comparison of 100 pM of synthetic target (TG) with 100 pM of RNA from selected probes where RNA was available. Multiple RNA extractions from different strains of the same species are shown in some cases (E16, 30 and 22 for probe 17 and E14 and 28 for probe 9).

A comparison of synthetic target vs. real RNA for several probes is shown in Figure 4. In many cases, the signal obtained from real RNA was at least 20× higher than that obtained with the synthetic target. The signal intensity difference between real RNA and synthetic target likely results from the greater thermodynamic stability of DNA/RNA heteroduplexes vs. DNA/DNA homoduplexes [27], the latter represents the synthetic target. Hybridising just below the melting temperature means that some targets will disassociate. The shorter the target (synthetic DNA), the easier it will be to disassociate. Although we did not fragment our RNA before hybridization, it is likely that in those cases where the signal from the RNA is lower than that from the synthetic target, the RNA was unwittingly fragmented during extraction and the target site was broken causing a lower signal to be obtained from the same amount of RNA or there was some inhibition from secondary structure formation. In one experiment where we did deliberately fragment the RNA to prevent secondary structure formation, we found no signal at all (data not shown). In an earlier study [28], we found that fragmentation of the RNA was required to prevent the secondary structure of the RNA from impeding probe binding. Because we are using much longer probes (>50 nts) as compared to the 22 nt in the Metfies et al. study, it is likely that the increased length of the probes and the higher annealing temperature have prevented secondary structure formation in the rRNA that could block probe binding. Thus, there is no need to fragment the RNA, which is interesting in terms of the simplicity of the technology and its future application at the point of care.

**Figure 5:**
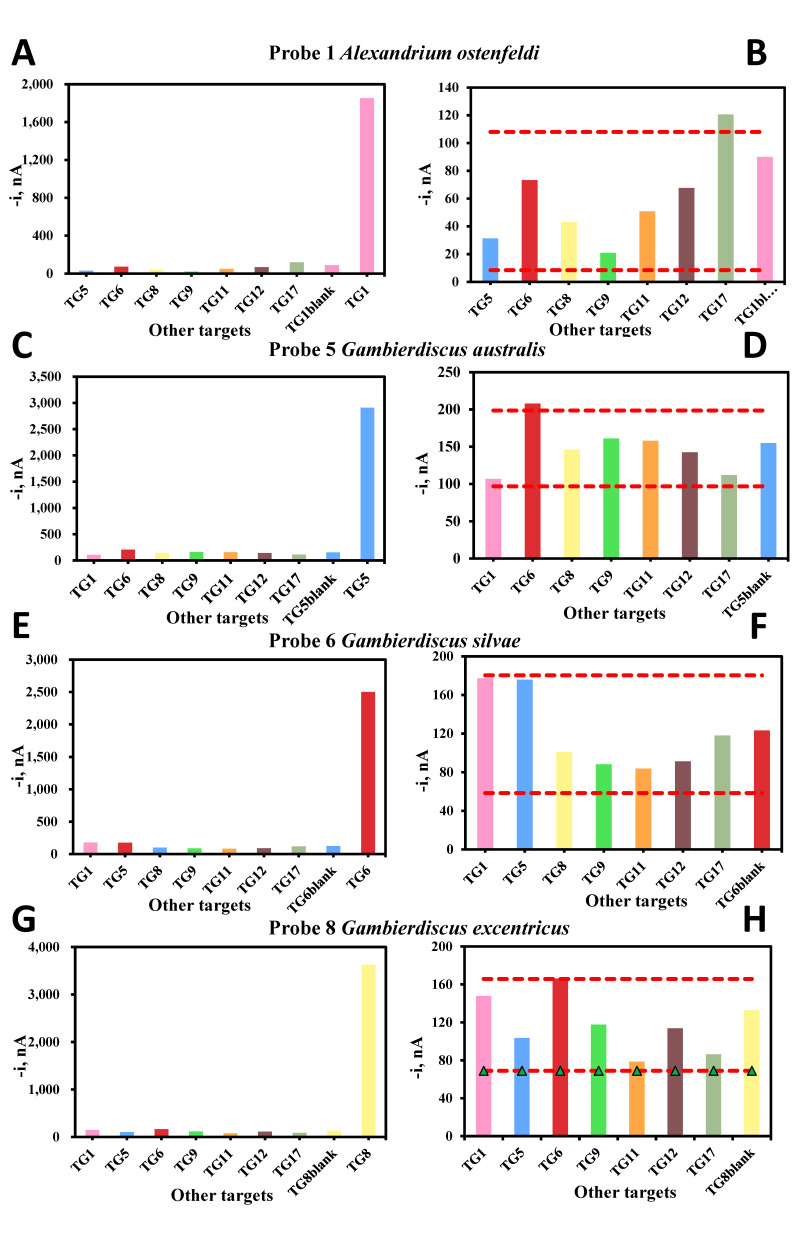
Comparison of cross-reactivity with synthetic targets (**A**,**C**,**E**,**G**) and non-targets (**B**,**D**,**F**,**H**) for *Alexandrium* and *Gambierdiscus* probes. +/− 1 × SD is plotted on the panels to the right to illustrate the signal difference between only non-targets and the blank (no target).

**Figure 6:**
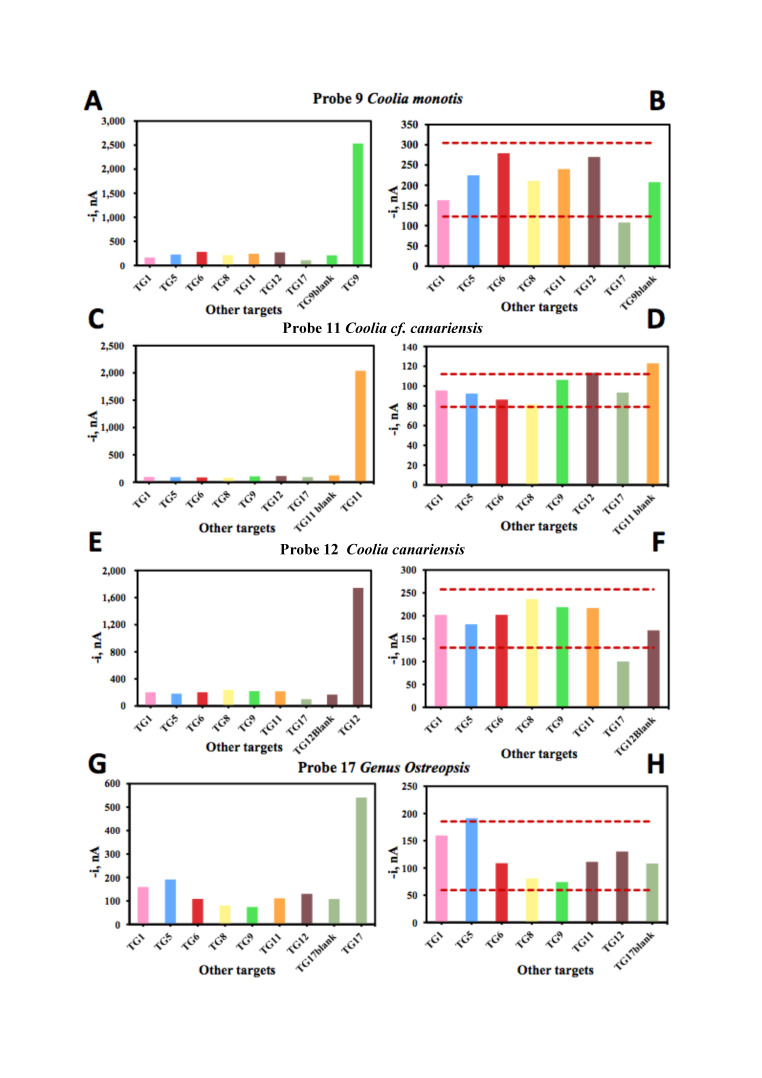
Comparison of cross-reactivity with synthetic targets (**A**,**C**,**E**,**G**) and non-targets (**B**,**D**,**F**,**H**) for *Coolia* and *Ostreopsis* probes. +/− 1 × SD is plotted on the panels to the right to illustrate the signal difference between only non-targets and the blank (no target).

Figure 5 and Figure 6 show the electrochemical signal for 100 pM of synthetic DNA target bound to the capture and signal probes for eight probes targeting *Gambierdiscus, Coolia, Ostreopsis*, *Lingulodinium* and *Alexandrium*. The positive target is plotted with the negative targets in the left panel of each probe set. In the panel to the right, ± 1 × SD is plotted for only the non-targets to show that the signals for the non-targets are less than 1 × SD from the blank. In each case, the positive synthetic target is over 200× stronger than the non-targets.

**Figure 7:**
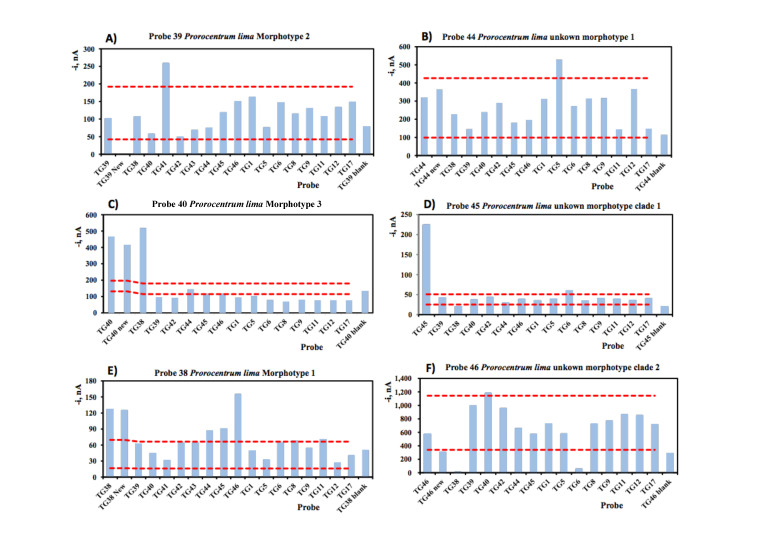
Comparison of cross-reactivity with synthetic targets and non-targets for *Prorocentrum lima* probes. +/− 1 × SD is plotted to illustrate the signal difference between non-targets and the blank (no target).

**Figure 8:**
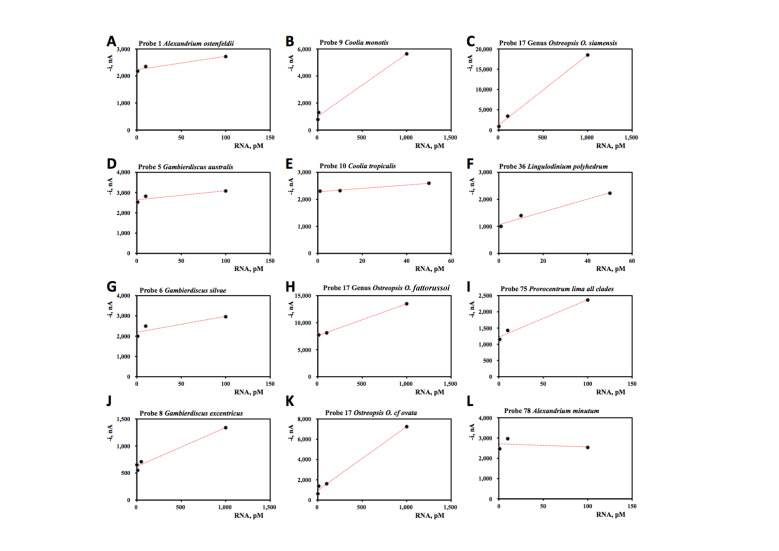
Concentration curves for probes where RNA was available. Regression statistics are present in Table 4 and cell counts inferred from 1 pM of RNA are presented in Table 3.

Of the ten probes designed for the ten unique clades of *Prorocentrum lima* in our ARB database, we tested 6 of them because European strains were recovered in these clades. As already shown in the in-silico tests for the cross-reactions of *Prorocentrum lima* probes, we expected their performance to be poorer than the probes shown in Figure 5 and Figure 6. Probes 38, 39, 40, 44 and 46 to different morphotypes of *Prorocentrum lima* were not very strong as compared to those in Figure 5 and Figure 6 and in each case, some non-targets were stronger than the target (Figure 7). The non-target signal was not strong enough to warrant the probes being renamed and used to target a different clade. Instead, we designed another probe (probe 75) to recognise all of the *Prorocentrum lima* clades. This probe recognises all the clades except *P. lima* unknown morphotype clade 1, which is recognised by probe 45 and fortunately is the strongest of all of the *Prorocentrum* probes tested, with almost no cross-reaction to the other clades (Figure 7D). These two probes, when used together, can be used to detect all *Prorocentrum lima* isolates known to date. Probe 75 was synthesized only as a combination of the signal and capture probe and was tested only with real RNA (Figure 8) because the blast search showed no cross-reactivity with the probes from the other species.

Concentration series were performed with those probes for which RNA was available (Figure 8). Because we found that many of the probes were saturated at 1000 pM (data not shown), we tested only the range 1, 10, 50 and 100 pM, occasionally 500 and 1000 pM. Good correlations between signal strength and RNA concentration (R^2^ > 0.90) were found for seven probes (probe 1: *Alexandrium ostenfeldii*, probe 8: *Gambierdiscus excentricus*, probe 9: *Coolia monotis*, probe 10: *Coolia tropicalis,* probe 17: Genus *Ostreopsis* (three strains), probe 36 *Lingulodinium polyhedrum*, probe 75: *Prorocentrum lima* all clades). Moderate correlations between signal strength and RNA concentration (R^2^ = 0.80–0.89) were found in two probes (probe 5: *Gambierdiscus australis*, probe 6: *Gambierdiscus silvae*). The only species so far tested that gave a poor correlation between the probe signal and RNA concentration was *Alexandrium minutum*. The signals obtained suggest that this probe (78) is saturated at 100 pM but this needs to be repeated with other RNA preps of this species. In several cases, we had multiple RNA preparations from different strains of the same species. Not all strains of the same species responded in a similar fashion but this could be an effect of the quality of the RNA extraction and whether or not the RNA was fragmented. The correlations likely varied with the quality of the RNA extraction and we present here the optimal correlation obtained.

A concentration series is needed for each probe to be able to determine how many cells are detected from any given probe signal. In this manner, the detection system can link with the alert levels for toxic algae in national monitoring programs. In Table 3, we present the number of cells equivalent to 1 pM of RNA. In most cases, 1 pM of RNA was near the detection limit of the method. In all cases, this was well below the maximum cell number needed to close the fisheries or close the beaches as a tourist alert, which is not always the same as in all countries (Table 3), especially because these species are not known in cold temperate climates. Given that our probes are to be used as an early warning system, monitoring would be conducted year-round and low concentrations of cells would be detected early in the season before blooms are initiated to allow mitigation strategies to be put into place as they rise.

Massive *Ostreopsis* spp. blooms have been detected in several samples from Caleta Caballo (Lanzarote) with up to 28,117 and 151,499 cells g^−1^ algal host plant [22] so our range of detection of 11-43 cells is well below the trigger levels recommended for *O.* cf. *ovata* (Table 3). If the genus has a taxonomic revision, we have already designed probes for all of the species/clades in the rRNA tree, which are ready to be tested if needed (Table 1).

In Catalonia, IRTA has alert levels for *Ostreopsis* for tourism but not for fisheries or aquaculture because the toxins from *Ostreopsis* and *Coolia* are not yet on the list of the official control for shellfish and thus no management decisions based on these two genera are taken in this part of the Mediterranean. Some beaches have been closed based on the abundance of *Ostreopsis* (Margarita Fernandez, IRTA, pers. comm.). In France, alert levels for *Ostreopsis* have changed over the years from being 30,000 cells L^−1^ in 2007/2008 to 100,000 cells L^−1^ in 2009 [20] but is now at 30,000 (Table 3). This will likely change in the near future as more and more intoxications occur but so far in Mediterranean waters, no deaths have occurred only irritations from aerosols.

In Diercks et al. [29], the electrochemical method used in that study could detect a minimum of 6250 cells of *A. minutum*, whereas, with our new improved protocol, we detected a minimum of 41 cells. This amounts to an improvement in sensitivity of >150×. This probe needs to be retested with different rRNA extractions because it appears to be saturated at 100pM, which makes its LOD likely even lower than the 41 cells we calculated, which is near the trigger level for that species (Table 3).

**Figure 9:**
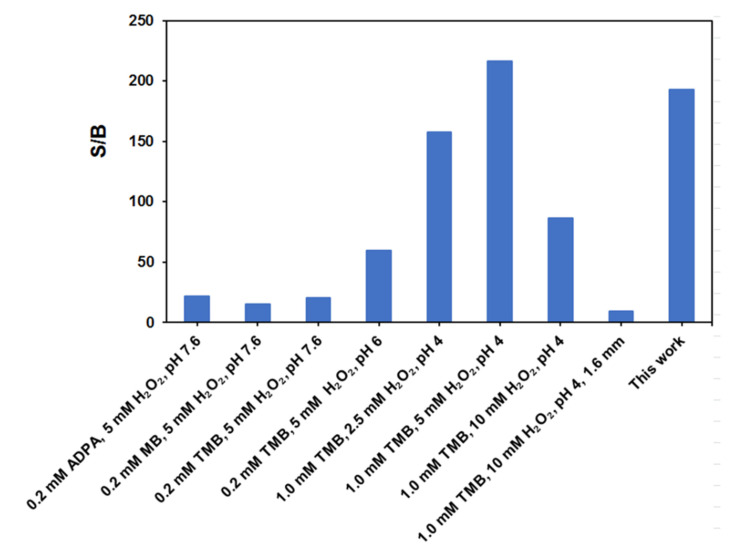
Comparison of the relationship between current density values obtained in the presence (1.0 μM) and absence of synthetic target DNA (S/B ratio) provided by the biosensors described in [28,29,30] for columns 1,2,3–8, respectively} for the determination of toxic algae changing mediators, labels, reagent concentrations and pH along with a final change to a single probe (this study).

The comparison with other electrochemical biosensors previously described for toxic algae (all of them also by Dr. Medlin) is also worth noting, as shown in Figure 9, comparing the relationship between current density values obtained in the presence (1.0 μM) and absence of synthetic target DNA (S/B ratio). The one described in this work offers an S/B close to the best (193 vs. 217) described in reference [30]. However, it is important to point out that besides using another redox mediator (methylene blue, MB, instead of HQ) and a 10-times higher concentration of enzymatic substrate (5 mM vs. 0.5 mM), the biosensor described in Ref. [30] requires 12−14 h for the determination against the 2 h of the one proposed in this work. Using other probes but similar hybridization conditions, bCp-MBs were demonstrated to be stable when stored at 4 °C in Eppendorf tubes containing 50 μL of filtered B&W buffer during at least 2 months [15] and the immobilisation of capture probes on the electrodes was stable at 4 °C for up to one year [29] thus confirming that immobilized probes are stable enough for commercialisation.

## 4. Conclusions

Oligonucleotide probes or barcodes can detect individual species or even strains of species. Detection of the target species takes place with a labelled barcode for the target species. We have shown here that our designed probes (barcodes) are highly specific and extremely sensitive, detecting as few as 10 cells. We are still testing probe specificity as we continue collecting RNA from target species.

To begin the detection using electrochemical signal amplification, sandwich DNA homohybrids or DNA/RNA heterohybrids are enzymatically labelled with HRP using appropriate commercial bioreagents. The variation in the cathodic current measured by amperometry, corresponding to the HRP reduction of H_2_O_2_ mediated by HQ, is proportional to the amount of the bound enzyme (and hence to the target concentration in a sample, i.e., the number of toxic algal cells as shown by our concentration series). Thus, barcodes (probes) are an effective molecular tool for monitoring toxic species. Although molecular methods are now widely used in environmental studies, they are permitted for monitoring purposes of toxic algae only in New Zealand. We are presently undertaking negotiations with the European Reference Laboratory to permit molecular monitoring in European waters because detection of species by DNA in a microarray format now has an ISO number (ISO 16578: 2013(en)) and thus, is now a fully accredited method for determining the concentration of DNA (= any species) in any environmental sample.

Thus, the electrochemical measurement methods presented here are a cheaper and easier alternative to light microscopic counting (compare EUR 2 per electrode and 2 h processing time to EUR 250 per sample and three to five days processing time). The fluorescent detection methods used in microarray hybridization, e.g., MIDTAL microarray, are equally fast (two to three hours but are prohibitively expensive EUR 250 per sample and EUR 65K for the fluorescent reader).

We are working on a new platform, a laboratory on a chip that will be universal, including all species on the MIDTAL microarray. The MIDTAL microarray has 60 probes for 33 species or combinations of species and we will design new longer probes for the MIDTAL species. When coupled with the probes tested here, our platform will be universal because probes for all known toxic species will be present. The MIDTAL microarray, now marketed by Microbia Environnement, does not contain these species and is therefore not a universal platform. The higher taxonomic probes on the MIDTAL microarray, required to ensure microarray specificity (24+ probes) in a hierarchical fashion, are not needed in the biosensor detection because our longer probes ensure higher specificity. Our planned platform is a multiplexed LOC, which represents the most cost-effective and sensitive method available for probe detection.

Moreover, our protocol used can easily be multiplexed and applied to other HABs detection methods by simply modifying the probes. This method is applicable to decentralized detection even in low-resource environments because of the characteristics of electrochemical detection with screen-printed electrodes. Although official closure of fisheries is based on toxin levels, the monitoring of cell numbers indicate when toxin monitoring should be increased and acts as an early warning system to both local gathers of shellfish and the aquaculture industry as a whole. Many countries follow these cell number triggers to initiate increased toxin sampling, and others, such as Spain, perform daily toxin tests and less frequent cell counts. [18,19,20,21]. Early warning systems provide a means to initiate mitigation strategies well in advance of any bloom to prevent huge economic losses from occurring.

## Figures and Tables

**Table 1:** List of probes designed EMERTOX and their target. Those marked in grey were tested here. C denotes the capture probe and S denotes the signal probe to detect the synthetic target. They were combined together as a single probe to detect RNA.

Probe Set No.	Probe Name	Target	Gene
1	AlOstS01C	*Alexandrium ostenfeldii*	SSU
	AlOstS01S		SSU
	AlOstS02C		SSU
	AlOstS02S		SSU
	AlOstS03C		SSU
	AlOstS03S		SSU
3	ClDinoS01C	Class Dinoflagellata	SSU
	ClDinoS01S		SSU
4	GaustD301C	*Gambierdiscus australis*	LSU D1-3
	GaustD301S		LSU D1-3
5	GaustD801C		LSU D8-10
	GaustD801S		LSU D8-10
6	GSilvD301C	*Gambierdiscus silvae*	LSU D1-3
	GSilvD301S		LSU D1-3
7	GExceD301C	*Gambierdiscus excentricus*	LSU D1-3
	GExceD301S		LSU D1-3
8	GExceD801C		LSU D8-10
	GExceD801S		LSU D8-10
9	CMonoS01C	*Coolia monotis*	SSU
	CMonoS01S		SSU
10	CtropD01C	*Coolia tropicalis*	LSU D1-3
	CtropD01S		LSU D1-3
11	CCanCd1D01C	*Coolia* cf. *canariensis*	LSU D1-3
	CCanCd1D01S		LSU D1-3
12	CCanCD2D01C	*Coolia canariensis*	LSU D1-3
	CCanCD2D01S		LSU D1-3
13	CspD01C	*Coolia* sp.	
	CspD01S		LSU D1-3
14	CPalmCld1D01C	*Coolia palmeriensis* Clade 1	LSU D1-3
	CPalmCld1D01S		LSU D1-3
15	C.palmCLD2D01C	*Coolia palmeriensis* Clade 2	LSU D1-3
	C.palmCLD2D01S		LSU D1-3
16	CooMalCD1D01C	*Coolia maliaensis*	LSU D1-3
	CooMalCD1D01S		LSU D1-3
16’	CooMalCD1D02C		LSU D1-3
	CooMalCD1D02S		LSU D1-3
17	GNOstreS01C	Genus *Ostreopsis*	LSU D1-3
	GNOstreS01S		LSU D1-3
18	Ostsp1D01C	*Ostreopsis* sp. 1	LSU D1-3
	Ostsp1D01S		LSU D1-3
19	Ostsp1D03C		LSU D1-3
	Ostsp1D03S		LSU D1-3
20	Ostsp2D01C	*Ostreopsis* sp. 2	LSU D1-3
	Ostsp2D01S		LSU D1-3
21	Ostsp3D01C	*Ostreopsis* sp. 3	LSU D1-3
	Ostsp3D01S		LSU D1-3
22	Ostsp4D01C	*Ostreopsis* sp. 4	LSU D1-3
	Ostsp4D01S		LSU D1-3
23	Ostsp5D01C	*Ostreopsis* sp. 5	LSU D1-3
	Ostsp5D01S		LSU D1-3
24	Ostsp6D01C	*Ostreopsis* sp. 6	LSU D1-3
	Ostsp6D01S		LSU D1-3
25	Ostsp7D01C	*Ostreopsis* sp. 7	LSU D1-3
	Ostsp7D01S		LSU D1-3
26	Ostsp8D01C	*Ostreopsis* sp. 8	LSU D1-3
	Ostsp8D01S		LSU D1-3
27	Ostsp9D01C	*Ostreopsis* sp. 9	LSU D1-3
	Ostsp9D01S		LSU D1-3
28	OstlentD01C	*Ostreopsis lentilosus*	LSU D1-3
	OstlentD01S		LSU D1-3
29	OstsiamD01C	*Ostreopsis siamensis*	LSU D1-3
	OstsiamD01S		LSU D1-3
30	OstrhodD01C	*Ostreopsis rhodensis*	LSU D1-3
	OstrhodD01S		LSU D1-3
31	OstovatD01C	*Ostreopsis ovata*	LSU D1-3
	OstovatD01S		LSU D1-3
	OstovatD02Competitor		LSU D1-3
32	OstCfOvCld2D01C	*Ostreopsis* cf. *ovata* clade 2	LSU D1-3
	OstCfOvCld2D01S		LSU D1-3
	OstCfOvCld2D02Comp		LSU D1-3
33	OstCf.AD01C	*Ostreopsis* sp. 10	LSU D1-3
	OstCf.AD01S		LSU D1-3
34	OstCf.BD8/03C	*Ostreopsis* sp. 11	LSU D1-3
	OstCf.BD8/03S		LSU D1-3
35	OstCf.DD07C	*Ostreopsis* sp. 12	LSU D1-3
	OstCf.DD07S		LSU D1-3
36	LinpolyD01C	*Lingulodinium polyheldrum*	LSU D1-3
	LinpolyD01S		LSU D1-3
37	VulrugD01C	*Vulcanodinium rugosum*	LSU D1-3
	Vul rugD01S		LSU D1-3
38	UnkPLMo1D01C	*Prorocentrum lima* morpho 1	LSU D1-3z
	UnkPLMo1D01S		LSU D1-3
39	UnkPLMo2D02C	*Prorocentrum lima* morpho 2	LSU D1-3
	UnkPLMo2D02S		LSU D1-3
40	UnkPlMo3D01C	*Prorocentrum lima* morpho 3	LSU D1-3
	UnkPlMo3D01S		LSU D1-3
41	UnkPlMo4D01S	*Prorocentrum lima* morpho 4	LSU D1-3
	UnkPlMo4D01C		LSU D1-3
42	UnkPlMo5D02C	*Prorocentrum lima* morpho 5	LSU D1-3
	UnkPlMo5D02S		LSU D1-3
43	unkMorC1D01C	*Prorocentrum lima* Unknown morphotype clade 1	LSU D1-3
	unkMorC1D01S		LSU D1-3
44	unkMorC1D02C		LSU D1-3
	unkMorC1D02S		LSU D1-3
45	unkMorC1D03C		LSU D1-3
	unkMorC1D03S		LSU D1-3
46	UnkMorC2D03C	*Prorocentrum lima* Unknown morphotype clade 2	LSU D1-3
	unkMorC2D03S		
75	PlimaD01C	*Prorocentrum lima* all clades	LSU D1-3
	PlimaD01S		LSU D1-3
76	UnkPLMo2D01C	*Prorocentrum lima* morpho 2	LSU D1-3
77	Plima?D01C	? *Prorocentrum* lima	LSU D1-3
78	AminD01*	*Alexandrium minutum*	LSU D1-3

**Table 2:** Absolute values for energy required to denature a double stranded helix. The energy required for probe to target is shown on the yellow diagonal (100% match between probe and target). Those figures shown in red represent potential cross-reaction and those in red and boxed, the non-target signal is close to or greater than the target and are cross-reactions. Probes for the clades of *Prorocentrum lima* are to the right of the vertical bar.

	1	5	6	8	9	11	12	17	38	39	40	41	42	43	44	45	46
	AO	GA	GS	GE	CM	CCD1	CCD2	GnOst	PLMo1	PLMo2	PlMo3	PlMo4	PlMo5	Mor1C1	Mor1C2	Mor1C3	Mo2C2
AO	−475.7	−60.5	−59.4	−61.6	−36.7	−54.9	−50.7	−48.6	−42.3	−31.3	−48	−50.6	−40.4	−47.3	−57.1	−44.3	−31.3
GA	−63.5	−470.2	−65.9	−39.5	−55.5	−60.8	−57.3	−52.2	−34.6	−40.2	−46.7	−64.4	−57.7	−63.6	−119.1	−56.5	−40.2
GS	−52.2	−48.7	−484.1	−98.6	−38	−49.1	−48.6	−63.3	−50.8	−37.1	−53.2	−51.9	−51.5	−54.6	−73.8	−50.1	−37.1
GE	−69.1	−52.9	−58.1	−472	−29.5	−35.7	−45.3	−69.4	−59.1	−31.3	−54.1	−56.2	−33.2	−41.4	−51.4	−50.7	−31.3
CM	−35.8	−53.3	−34.6	−50.7	−422.6	−38.7	−35.2	−87.1	−60.4	−87.9	−38.3	−44.1	−31	−30.6	−64.4	−70.2	−87.9
Ccf.Can	−38.7	−39.6	−30.7	−51.6	−39.1	−303.8	−77.3	−40.4	−60.8	−33	−46.7	−49.2	−83.3	−52.2	−52.8	−43.6	−33
CCan	−50.1	−57.8	−48.6	−44.2	−42	−48.7	−524.6	−55.1	−44.1	−34.3	−46.4	−44.1	−58.3	−74.6	−53.5	−80.8	−34.3
GnOst	−49.4	−69.8	−42.4	−77.3	−36.9	−58.3	−53.9	−328.3	−46.5	−60	−130.6	−61.6	−33.1	−59.3	−55.2	−46.2	−60
PLMo1	−60.2	−79.9	−36	−66.7	−65.4	−82	−39.8	−51.9	−389.1	−36.1	−58.9	−269.8	−80.4	−40.1	−115.2	−47.2	−36.1
PLMo2	−43	−35.3	−91.5	−36.7	−77.6	−41.3	−31.5	−111.6	−44.7	−392.9	−149.9	−49.9	−70	−64.4	−53.6	−51.9	−381.2
PlMo3	−47.2	−48.8	−49.8	−45.1	−41.5	−65.1	−49	−79.3	−84.2	−118.2	−467.8	−61.3	−45.3	−56.2	−55.4	−138.3	−114.9
PlMo4	−53.3	−36.8	−55.9	−39.9	−46.4	−51.5	−42.5	−71.1	−281.6	−65	−58.9	−384.1	−54.8	−57.4	−61.3	−44.8	−65
PlMo5	−45.8	−40.3	−26.8	−74.6	−27	−85.5	−90.3	−53	−44.2	−69	−58.6	−44.2	−463.2	−291	−41.4	−79.1	−69
Mor1C1	−45.8	−56.5	−51.3	−47	−37	−137.1	−114.9	−53	−50.9	−50.5	−115.8	−69.1	−283.5	−452	−41.4	−54.7	−50.5
Mor1C2	−49.1	−94.4	−66.1	−62.3	−69.7	−30.3	−55.6	−93.2	−136.4	−82.3	−48	−51.3	−46.4	−46.4	−391.1	−41.7	−82.3
Mor1C3	−24.5	−45.2	−44.8	−69.5	−38.5	−67.5	−77.3	−40	−51.3	−34.3	−125.9	−38.9	−27.7	−27.7	−44.9	−416.3	−34.3
Mo2C2	−43	−35.3	−91.5	−36.7	−77.6	−41.3	−31.5	−111.6	−60.1	−382.5	−139.5	−49.9	−70	−64.4	−53.6	−28.2	−378.6

**Table 3:** Estimation of the number of cells equivalent to 1 pM of RNA as compared to the number required to trigger a warning. Alerts are usually about 1/3 of the level as a warning. Trigger levels were taken from [18,19,20,21].

Species/Strain from IEO VIGO and AWI	Number of Cells for 1 pM of RNA	Maximum Number of Cells Allowed (Per L) Before Fisheries Are Closed or More Intensive Toxin Testing is Required or Beaches Closed to Swimming
*Prorocentrum lima/*2V	444	100 (UK) 500 (Spain)
*P. lima/*PL27V	154	100 (UK) 500 (Spain)
*P. lima/*PL7V	254	100 (UK) 500 (Spain)
*P. lima*/PLMA01	10	100 (UK) 500 (Spain)
*Lingulodinium*/1204	142	10,000 (0.000 (France))
*Coolia sp./*VGO782	34	N/A
*Coolia sp.*/941	32	N/A
*Coolia malayensis*/1163	117	N/A
*Coolia monotis*/CMIV	19	N/A
*Coolia monotis*/VGO831	174	N/A
*Coolia tropicalis*/923	57	N/A
*Ostreopsis cf ovata*/1107	26	10-30,000 Italy ^1^, France
*Ostreopsis cf ovata*/614	11	10–30,000 Italy, France
*Ostreopsis cf ovata*/820	43	10–30,000 Italy, France
*Ostreopsis cf ovata*/884	18	10–30,000 Italy, France
*Ostreopsis cf ovata*/1196	14	10–30,000 Italy, France
*Ostreopsis cf ovata*/1068	33	10–30,000 Italy, France
*Ostreopsis cf ovata*/898	22	10–30,000 Italy, France
*Ostreopsis* cf. *ovata*/693	12	10–30,000 Italy, France
*Ostreopsis fattorussoi*/1795	45	10–30,000 Italy, France
*Alexandrium ostenfeldii*	56	40 (UK), 200 (Aus)
*Alexandrium minutum*	41	Presence (Northern Europe), 40–1000 (Southern Europe), 200 (Aus)

^1^ With wind conditions producing aerosols [20].

**Table 4:** Analytical characteristics of the calibration plots displayed in Figure 8.

Probe Number	Species Name	Slope, pM nA^−1^	Intercept, nA	R^2^
1	*Alexandrium ostenfeldii*	4.9 ± 1.1	2233 ± 69	0.94
5	*Gambierdiscus australis*	4.5 ± 2.3	2642 ± 134	0.88
6	*Gambierdiscus silvae*	7.9 ± 3.9	2194 ± 227	0.80
8	*Gambierdiscus*	0.72 ± 0.08	621 ± 41	0.98
9	*Coolia monotis*	4.6 ± 0.4	1014 ± 240	0.99
10	*Coolia tropicalis*	6.2 ± 0.8	2280 ± 22	0.99
17	Genus *Osteopsis, O. fattorussoi*	5.9 ±0.1	7599 ± 79	0.99
	Genus *Ostreopsis, O.* cf. *ovata*	6.3 ± 0.4	968 ± 209	0.99
	Genus *Ostreopsis, O. siamensis*	17 ± 1	1270 ± 432	0.99
36	*Lingulodinium polyhedrum*	23.7 ± 3.6	1059 ± 107	0.99
75	*Prorocentrum lima* all clades	11 ± 2	1221 ± 93	0.98
78	*Alexandrium minutum*	−1.5 ± 4.6	2713 ± 273	0.096
